# Attenuative Effects of Fluoxetine and *Triticum aestivum* against Aluminum-Induced Alzheimer’s Disease in Rats: The Possible Consequences on Hepatotoxicity and Nephrotoxicity

**DOI:** 10.3390/molecules26216752

**Published:** 2021-11-08

**Authors:** Karema Abu-Elfotuh, Ghada M. Ragab, Ahmad Salahuddin, Lubna Jamil, Ekram Nemr Abd Al Haleem

**Affiliations:** 1 Department of Pharmacology and Toxicology, Faculty of Pharmacy, Al-Azhar University, Cairo 11754, Egypt; karimasoliman.pharmg@azhar.edu.eg (K.A.-E.); ekramnemr.pharmg@azhar.edu.eg (E.N.A.A.H.); 2 Department of Pharmacology and Toxicology, Faculty of Pharmacy, Misr University for Science and Technology, Giza 12585, Egypt; ghada.ragab@must.edu.eg; 3 Department of Biochemistry, Faculty of Pharmacy, Damanhour University, Damanhour 22511, Egypt; 4 Department of Histology, Faculty of Medicine, October 6 University, Giza 12585, Egypt; lubnajamil.med@o6u.edu.eg

**Keywords:** Alzheimer’s, *Triticum aestivum*, β–catenin, GSK-3β, β-amyloid, tau protein, BDNF, hepatotoxicity, nephrotoxicity

## Abstract

Background: Alzheimer’s disease (AD) is a chronic neurological illness that causes considerable cognitive impairment. Hepatic and renal dysfunction may worsen AD by disrupting β-amyloid homeostasis at the periphery and by causing metabolic dysfunction. Wheatgrass (Triticum aestivum) has been shown to have antioxidant and anti-inflammatory properties. This work aims to study the effect of aluminum on neuronal cells, its consequences on the liver and kidneys, and the possible role of fluoxetine and wheatgrass juice in attenuating these pathological conditions. Method: Rats were divided into five groups. Control, AD (AlCl_3_), Fluoxetine (Fluoxetine and AlCl_3_), Wheatgrass (Wheatgrass and AlCl_3_), and combination group (fluoxetine, wheatgrass, and AlCl_3_). All groups were assigned daily to different treatments for five weeks. Conclusions: AlCl_3_ elevated liver and kidney enzymes, over-production of oxidative stress, and inflammatory markers. Besides, accumulation of tau protein and Aβ, the elevation of ACHE and GSK-3β, down-regulation of BDNF, and β–catenin expression in the brain. Histopathological examinations of the liver, kidney, and brain confirmed this toxicity, while treating AD groups with fluoxetine, wheatgrass, or a combination alleviates toxic insults. Conclusion: Fluoxetine and wheatgrass combination demonstrated a more significant neuroprotective impact in treating AD than fluoxetine alone and has protective effects on liver and kidney tissues.

## 1. Introduction

With increased human life expectancy, dementia constitutes one of the most significant social, economic, and public health issues. According to an epidemiologic survey, around 50 million people worldwide had dementia in 2018, with Alzheimer’s disease (AD) accounting for about 60 to 80% of all cases. The percentage is set to triple by 2050 [1]. Increased age is the most critical risk factor for AD development [2]. Family history [3], degeneration or vascular dysfunction [4], overweight [5], hypotension or hypertension [6], diabetes [7], hyperlipidemia [8], low levels of education, and lack of physical activity [9] are all realized risk factors.

AD is a neurodegenerative illness; its leading cause is neuronal cell death. AD is marked by pathophysiological abnormalities in the brain. One of these abnormalities is the accumulation of beta-amyloid (Aβ) inside the neurons, which may contradict acetylcholine’s ability to influence synaptic transmission and initiate inflammatory processes [10].

In the senile dementia of Alzheimer’s type, the decline of acetylcholine levels may be due to a reduction in choline acetyltransferase levels, the enzyme involved in acetylcholine synthesis. In turn, the loss of acetylcholine was reported to be associated with the production of Aβ [11]. Aβ plays a central role in producing the cholinergic deficit, as it reduces acetylcholine synthesis. Furthermore, some evidence also suggests the involvement of acetylcholine esterase in the pathogenesis of AD, as acetylcholine esterase interacts with the Aβ peptide and promotes amyloid fibril formation [11].

Additionally, the accumulation of Aβ leads to oxidative stress and inflammation in the AD brain and, thereby, neurodegeneration. As a result, reactive oxygen species (ROS) form free radicals that attack the cell membrane, mitochondria, lipids, and proteins, causing neuronal cell apoptosis. The inflammation produces cytokines by activation of the microglia and inhibits the production of brain-derived neurotrophic factor (BDNF), which exerts neuronal protection, synaptogenesis, and neurogenesis [12]. In effect, neuroinflammation is responsible for an abnormal secretion of proinflammatory cytokines which trigger signaling pathways that activate brain tau hyperphosphorylation in residues that are not modified under normal physiological conditions [13]. The hyperphosphorylation of tau protein may form neurofibrillary tangles (NFTs). Consequently, this may lead to blockage of neurotransmitters and thus neuronal cell death [10].

The liver is the primary organ that metabolizes more than 60% of Aβ [14]. Eliminating circulating Aβ may hasten AD development by shifting the dynamic balance away from Aβ accumulation in senile plaques toward soluble Aβ. Decreased liver metabolism could lead to brain Aβ accumulation [15]. So, hepatic dysfunction may play a role in AD through the inability to maintain Aβ homeostasis at the periphery, acting as a source of proinflammatory cytokines and metabolic dysfunction [16]. In addition, novel dementia medications could target decreased hepatic synthesis or greater peripheral clearance of Aβ protein.

Chronic kidney disease (CKD) and AD are common chronic diseases in elderly communities and civilizations. CKD was found to be associated with dementia, as there is a high possibility of cognitive impairment or AD-like dementia in CKD patients. The kidney has a vital role in the peripheral clearance of Aβ. The vulnerability of brain tissue to vascular dysfunction, inflammation, oxidative stress, and the renin-angiotensin system may explain the cognitive loss and AD seen in CKD patients. Additionally, small vessel injury may play a non-negligible role in contributing to cognition impairment in both CKD and AD [17,18].

Fluoxetine, as a selective serotonin reuptake inhibitor (SSRI) antidepressant, could be used to relieve depression and anxiety among AD patients. Moreover, fluoxetine could improve memory and cognitive function for patients with mild cognitive impairment, an early AD state [19]. Furthermore, fluoxetine has been shown to inhibit β-amyloid production, prevent neuronal degeneration [20,21,22], and enhance the phosphorylation of GSK3β [23]. Besides, fluoxetine could potentially treat Alzheimer’s disease through the activation of Wnt/β-catenin signaling [24].

Beneficial food ingredients have been investigated for use in the treatment of AD patients to enhance memory and cognitive function [25]. Wheatgrass (common wheat) is the freshly sprouted first leaves of *Triticum aestivum* Linn, family Gramineae [26]. Wheatgrass is the earlier grass of wheat that is obtained on the eighth or ninth day after cultivation. Wheatgrass contains minerals, amino acids, vitamins C, E, and ß-carotene, and high content of chlorophyll. Furthermore, wheatgrass contains different phytochemicals such as alkaloids, flavonoids, phenolics, tannins, and glycosides. The antioxidant activity of wheatgrass is due to phenolics, which help to reduce the effects of diseases such as cardiovascular diseases, inflammation, and cancers [27,28,29].

Our study aimed to investigate the effect of aluminum on mental health and its consequences on the liver and the kidneys and the possible role of wheatgrass juice alone or in combination with fluoxetine in attenuating these effects.

## 2. Results

### 2.1. Behavioral Test (Morris Water Maze (MWM)

The AD group showed that the escape latency was longer than the control group by 127.4%. The AD group that received fluoxetine, wheatgrass, or their combination showed a significant decrease in the escape latencies time by 46.4%, 34.3%, and 47.1%, respectively, compared to the AD group. Comparisons showed that the escape latencies in the fluoxetine group were shorter than those in the wheatgrass group on days 3 and 4 (*p* = 0.002). The escape latency was the quickest in the group treated with the combination. Additionally, the AD group showed a shorter time spent in the target quadrant than that of the control group by 66.5%, but groups treated by fluoxetine, wheatgrass, or their combination showed a significant increase in the time spent in the target quadrant by 104.7%, 130.5%, and 171.7%, respectively, as compared to the AD group. However, groups treated by wheatgrass produced an apparent increase in the time spent in the target quadrant compared to fluoxetine treatment. Notably, the combination of fluoxetine and wheatgrass showed a marked increase in the time spent in the target quadrant compared to the wheatgrass group (Table 1).

### 2.2. Effect of Fluoxetine, Wheatgrass, or Their Combination on Alanine Transaminase (ALT), Aspartate Transaminase (AST), and Alkaline Phosphatase (ALP)

The AD group showed a significant elevation in ALT, AST, and ALP levels by 592.3, 336.1, and 225.3%, respectively, compared to control values. On the other hand, treatment by fluoxetine, wheatgrass, or their combination induced a significant decrease in ALT level by 27.8, 59.4, and 68.6%, respectively, compared to the AD group. Also, groups that received these treatments showed a significant reduction in AST level by 41.9, 53.4, and 65.4%, respectively, compared to the AD group. ALP levels also decreased in groups that received these treatments by 46.7, 50.1, and 55.4%, respectively, compared to the AD group (Table 2).

### 2.3. Effect of Fluoxetine, Wheatgrass, or Their Combination on Total Cholesterol (TC), Triacylglycerol (TG), and High-Density Lipoprotein (HDL)

The AD group showed a significant increase in TC and TG by 127.5 and 87.6%, respectively, compared to control values. The AD group treated by fluoxetine, wheatgrass, or a combination showed a significant decrease in TC level by 31.7, 41.8, and 43%, and TG by 37, 120.8, and 158%, respectively, compared to the AD group. However, groups treated with wheatgrass produced an apparent decrease in TC and TG compared to fluoxetine treatment. Remarkably, the combination of fluoxetine and wheatgrass showed a marked decline in these parameters compared to wheatgrass groups. In contrast, the AD group showed a significant decrease in HDL level by 70.3% compared to the control group. While, groups that received fluoxetine, wheatgrass, or their combination, showed a significant increase in HDL level by 113.8, 120.8, and 158%, respectively, compared to the AD group. Notably, the combination of fluoxetine and wheatgrass showed a marked increase in the HDL level compared to the other treated groups (Table 2).

### 2.4. Effect of Fluoxetine, Wheatgrass, or Their Combination on Hepatic Interleukin-6 (IL-6), Tumor Necrosis Factor-A (TNF-A), Nuclear Factor Kappa B (NF-Κb), and Caspase-3 Activity

The AD group showed a significant increase in hepatic IL-6, TNF-α, NF-κB levels, and Caspase-3 activity by 319.3, 270, 889.3, and 154.9%, respectively, compared to the control value. In contrast, the AD group that received fluoxetine, wheatgrass, or their combination showed a significant decrease in the hepatic IL-6 levels by 31.5, 40.9, and 53.2%, TNF-α levels by 18.6, 30.8, and 47.8%, NF-κB levels by 37.9, 52.9, 76.12%, and caspase-3 activity by 26.4, 44.9, and 46.2%, respectively, when compared to the AD group. In contrast, the group treated with wheatgrass significantly decreased these parameters compared to groups that received fluoxetine. Notably, the combination of fluoxetine and wheatgrass showed a marked decline in the inflammatory markers and apoptosis marker compared to other treated groups (Table 3).

### 2.5. Effect of Fluoxetine, Wheatgrass, or Their Combination on Hepatic Total Antioxidant Capacity (TAC), Superoxide Dismutase (SOD), Malondialdehyde (MDA), and Nitric Oxide (NO)

The AD group showed a significant increase in hepatic MDA and NO by 832.4 and 1143.1%, respectively, compared to the control values. Whereas treatment of the AD group by fluoxetine, wheatgrass, or a combination provided significantly lower MDA levels by 15.5, 49.9, and 72.4%, respectively, and NO levels by 38.7, 60.8, and 69.9%, respectively, compared to the AD group. However, groups treated with wheatgrass showed an apparent decrease in MDA and NO compared to groups that received fluoxetine. The combination of treatments produced a significant reduction in these parameters as compared to wheatgrass groups.

However, the hepatic SOD and TAC levels were significantly decreased by 86.6 and 66.6%, respectively, in the AD group compared with the control group. AD group received fluoxetine, wheatgrass, or their combination showed a significant increase in the hepatic SOD levels by 157.9, 317.7, and 462%, respectively, and TAC level by 60.4, 27.4, and 128.2%, respectively, when compared to the AD group. However, wheatgrass produced a significant elevation of SOD as compared to the fluoxetine group while the group that received fluoxetine showed a significant increase in TAC as compared to the wheatgrass group. Notably, the combination of fluoxetine and wheatgrass showed a marked increase in the antioxidant markers compared to the other treated groups (Table 3).

### 2.6. Effect of Fluoxetine, Wheatgrass, or Their Combination on Serum Creatinine and Urea

Compared to control values, the AD group showed a significant increase in serum creatinine and urea levels by 1292 and 99.8%. However, groups treated with fluoxetine, wheatgrass, or a combination resulted in a significant decrease in creatinine level by 29.3, 55.4, and 81%, respectively, and urea level by 29.5, 45.2, and 49.6%, respectively, when compared to the AD group. Moreover, wheatgrass produced a significant decrease in creatinine and urea levels when compared to the fluoxetine values. Notably, the combination of fluoxetine and wheatgrass showed a marked decrease in creatinine and urea compared to the wheatgrass group (Table 2).

### 2.7. Effect of Fluoxetine, Wheatgrass, or Their Combination on Renal Total Antioxidant Capacity (TAC), Superoxide Dismutase (SOD), Malondialdehyde (MDA), and Nitric Oxide (NO)

The AD group showed a significant increase in renal MDA and NO by 568.5 and 1139.3%, respectively, compared to the control values. Whereas treatment of the AD group by fluoxetine, wheatgrass, or a combination provided significantly lower MDA levels by 39, 62.5, and 75.1%, respectively, NO levels by 30.2, 55.9, and 74.3% respectively, compared to the AD group. On the other hand, groups treated with wheatgrass showed a significant decrease in MDA and NO compared to groups treated with fluoxetine. The combination of these treatments produced a significant decline in these parameters compared to the wheatgrass group.

Moreover, the renal SOD and TAC levels were significantly decreased by 88.1 and 58.4%, respectively, in the AD group compared with the control. The AD group that received fluoxetine, wheatgrass, or a combination showed a significant increase in the SOD levels by 180.4, 230.2, and 382.2%, and TAC levels by 35.6, 52.07, and 77.3%, respectively, when compared to the AD group. Moreover, wheatgrass produced a significant increase in TAC and SOD levels when compared to the fluoxetine values. Notably, the combination of fluoxetine and wheatgrass showed a marked increase in the antioxidant markers compared to the wheatgrass group (Table 4).

### 2.8. Effect of Fluoxetine, Wheatgrass, or Their Combination on Renal Interleukin-6 (IL-6), Tumor Necrosis Factor-A (TNF-A), Nuclear Factor Kappa B (NF-Κb), and Caspase-3 Activity

The effect of fluoxetine, wheatgrass, or their combination on renal IL-6, TNF, and NF-κB were assessed as markers of inflammation; also, caspase-3 activity was evaluated as a marker of apoptosis is shown in Table 4. Their levels were significantly elevated by 195.7, 272.5, 355.3, and 573.3%, respectively, in the AD group compared to the control group. The AD group treated by fluoxetine, wheatgrass, or their combination significantly decreased the elevation of IL-6 levels by 32.8, 43.1, and 47.9%, TNF-α levels by 37.4, 37.2, and 46.9%, NF-κB levels by 32.3, 36.2, and 42.6%, and caspase-3 activity by 58.7, 58 and 63.3%, respectively, compared to the AD group. Whereas the group treated with wheatgrass showed a significant decrease in IL-6 and NF-κB as compared to groups treated with fluoxetine. Notably, the combination of fluoxetine and wheatgrass showed a marked decline in the inflammatory markers and apoptosis marker compared to other treated groups (Table 4).

### 2.9. Effect of Fluoxetine, Wheatgrass, or Their Combination on Cerebral Β-Catenin and Glycogen Synthase Kinase-3 Beta (GSK-3β)

The AD group showed a significant decrease in cerebral β-catenin content by 80.68% compared to the control values. In contrast, the AD group that received fluoxetine, wheatgrass, or their combination showed a significant increase in the β -catenin levels by 189.11, 306.5, 408.02%, respectively, when compared to the AD group. While the AD group that received wheatgrass showed a significant increase in β-catenin levels compared to the fluoxetine values. Notably, the combination treatment resulted in a remarkable elevation in β-catenin as compared to wheatgrass values.

Besides, the AD group showed a significant increase in GSK-3β content by 900% compared to the control group. On the other hand, the effect of treatment by fluoxetine, wheatgrass, or their combination resulted in a significant decrease in the GSK-3β level by 42.85, 44.16, and 26.91% correspondingly when compared to the AD group. Additionally, there is no significant difference between groups treated with fluoxetine or wheatgrass. However, the combination of fluoxetine and wheatgrass showed a significant decline in GSK-3β levels compared to groups treated separately (Table 5).

### 2.10. Effect of Fluoxetine, Wheatgrass, or Their Combination on Cerebral Total Antioxidant Capacity (TAC), Superoxide Dismutase (SOD), and Malondialdehyde (MDA)

The data in Table 5 showed a significant decrease in cerebral TAC and SOD levels in the AD group by 72.05 and 91.07%, respectively, compared to the control group. While treatment of the AD group with fluoxetine, wheatgrass, or their combination produced a significant increase in cerebral TAC levels by 102.5, 93.5, 143.6%, and SOD levels by 451.8, 617.1, and 761.3%, respectively, in comparison with the AD group. Besides, groups treated with wheatgrass showed a significant increase in SOD compared to the fluoxetine group. Notably, the combination of fluoxetine and wheatgrass showed a marked elevation in SOD and TAC compared to the fluoxetine or wheatgrass groups.

Also, Table 5 showed that a significant increase in MDA level by 1432.3% as compared to the control group, but the treatment of AD with fluoxetine, wheatgrass, or their combination resulted in a significant decrease in the MDA level by 63.6, 54.2, and 67.9%, respectively, when compared to the AD group. Moreover, fluoxetine produces a significant reduction in MDA when compared to the wheatgrass values. Notably, the combination of fluoxetine and wheatgrass showed a marked decline in MDA values compared to the fluoxetine group (Table 5).

### 2.11. Effect of Fluoxetine, Wheatgrass, or Their Combination on Cerebral Neurotransmitters Dopamine (DA), Norepinephrine (NE), and Serotonin (5-HT)

The AD group showed a significant decrease in neurotransmitters DA, NE, and 5-HT by 76.1, 67.2, 64.8%, respectively, compared to the control group. However, groups treated with fluoxetine, wheatgrass, or their combination showed a significant increase in DA levels by 138.6, 74.1, 177.9%, 5-HT levels by 122.2, 68.7, and 158.9%, and NE levels by 147.4, 91.1, and 150.4%, respectively, as compared to the AD group. Besides, fluoxetine significantly increases DA, 5-HT, and NE compared to the wheatgrass values. Notably, the combination of fluoxetine and wheatgrass showed a tremendous increase in neurotransmitters compared to the fluoxetine group (Table 5).

### 2.12. Effect of Fluoxetine, Wheatgrass, or Their Combination on Cerebral Interleukin-1β (IL-1β) and Tumor Necrosis Factor-A (TNF-α)

Table 5 showed a significant increase in cerebral IL-1β and TNF-α in the AD group by 318 and 684.9%, respectively, compared to the control group. Moreover, groups treated with fluoxetine, wheatgrass, or a combination showed a significant decrease in IL-1β by 51.1, 29.3, and 54.1%, and TNF-α level by 70.2, 58.7, and 71.9%, respectively, when compared to the AD group. However, groups treated with fluoxetine produced a clear decline in cerebral proinflammatory IL-1β and TNF-α as compared to groups that received wheatgrass, while the combination of treatments showed a significant decrease in these parameters more than fluoxetine.

### 2.13. Effect of Fluoxetine, Wheatgrass, or Their Combination on Cerebral Beta-Amyloid (Aβ), Tau Protein (TAU), Acetylcholine Esterase (ACHE), and Brain-Derived Neurotrophic Factor (BDNF)

The AD group showed a significant increase in cerebral AB contents, TAU level, and ACHE activity by 2046.9, 900.9, and 413.5%, respectively, compared to the control values. Moreover, groups treated with fluoxetine, wheatgrass, or their combination showed a significant decrease in AB contents by 52, 43.9, and 74.8%, TAU level by 56.1, 42.8, and 62.9%, and ACHE activity by 50, 31.4, and 69.3%, respectively, when compared to the AD group. Moreover, fluoxetine significantly decreases AB, TAU, and ACHE levels compared to the wheatgrass values. Conspicuously, the combination of fluoxetine and wheatgrass showed a clear decline in these markers compared to the fluoxetine group.

**Figure 1:**
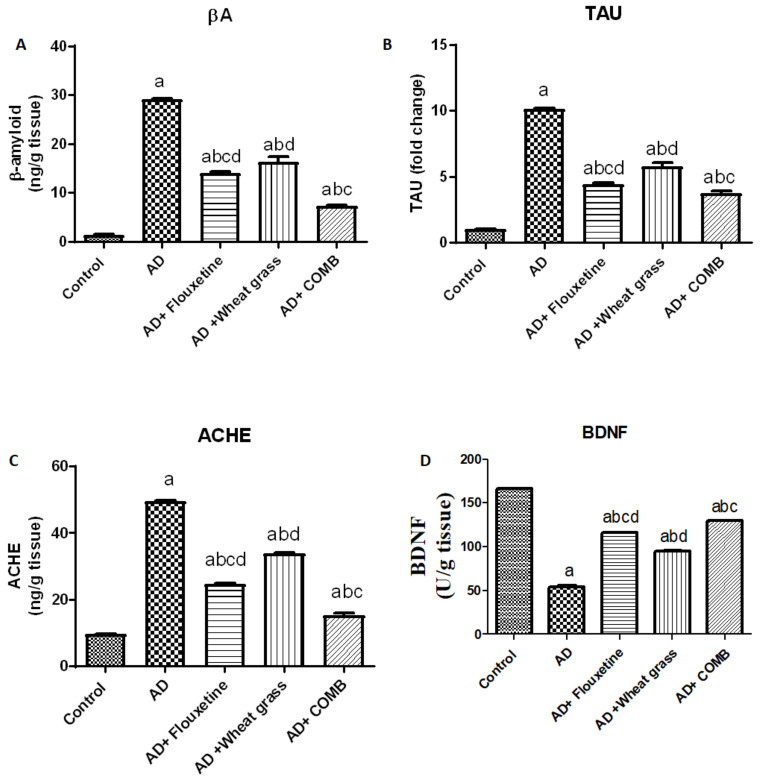
Effect of fluoxetine, wheatgrass, or their combination on cerebral Aβ (**A**), TAU (**B**), ACHE (**C**), and BDNF (**D**), Data were expressed as means ± SD. a, b, c, or d. Significantly different from the control, AD, wheatgrass/AD or combination/AD group, respectively, *p* < 0.05 using ANOVA followed by Tukey–Kramer as post hoc test.

In contrast, the AD group showed a significant decline in BDNF contents by 67.1% compared to the control values. Likewise, groups treated with fluoxetine, wheatgrass, or their combination showed a significant increase in BDNF contents by 112.9, 73.4, and 138.8%, respectively, when compared to the AD group as shown in Figure 1D. However, groups treated with fluoxetine showed a significant decline in BDNF compared to groups that received wheatgrass. The combination of treatments produced the most significant decline in BDNF (Figure 1).

### 2.14. Effect of Fluoxetine, Wheatgrass, or Their Combination on Histopathological Examination of The Liver, Kidney, and Brain Tissue Specimens

**Figure 2:**
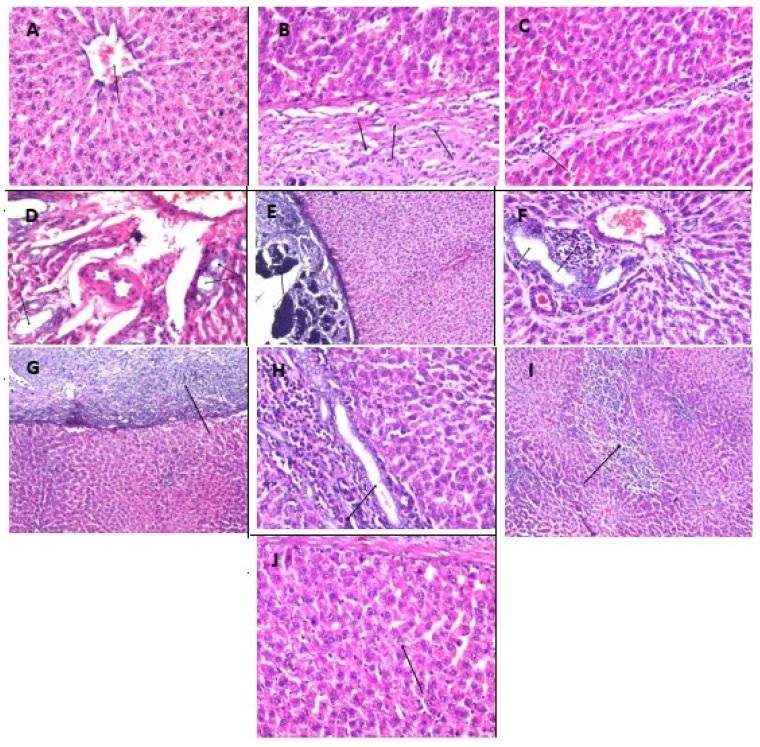
Photomicrographs of hepatic tissue specimens stained by H & E (×40). Photomicrograph (**A**) Transverse hepatic tissue section from the control group showing the histological structure of the central vein and surrounding hepatocytes in the parenchyma. Photomicrographs (**B**–**D**) Transverse hepatic tissue section from AlCl_3_-treated animals showing the hepatic capsule was thick due to fibrous connective tissue proliferation and inflammatory cell infiltration (**B**), strands of fibrous tissue formation with inflammatory cells infiltration were extended in between the hepatocytes (**C**). The portal area showed congestion in the portal vein with multiple newly formed bile ductules (**D**) (arrows). Photomicrographs (**E**,**F**): Transverse hepatic tissue section from the fluoxetine-treated animals showing Glisson’s capsule with fibrosis and inflammatory cells infiltration as well as calcification (**E**) associated with inflammatory cells infiltration in the portal area (**F**) (arrows). Photomicrographs (**G**–**I**)**:** Transverse hepatic tissue section from the wheatgrass group showing Glisson’s capsule with fibrosis, thickening, and inflammatory cells infiltration (**G**), while the portal area had hyperplasia in the bile ducts with inflammatory cells infiltration in between (**H**). There was focal necrosis in the parenchyma (**I**) (arrows). Photomicrograph (**J**): Transverse hepatic section from the combination group showing no histopathological alteration.

**Figure 3:**
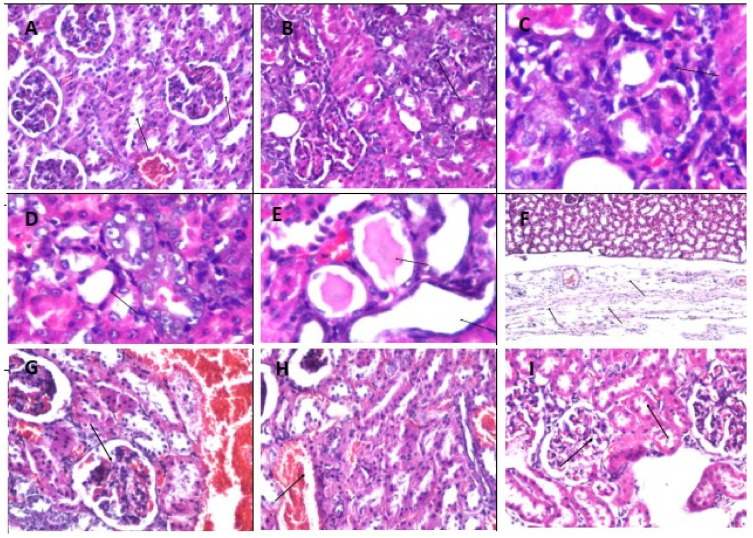
Photomicrographs of renal tissue specimens stained by H & E (×40). Photomicrograph (**A**): Transverse renal tissue section from the control group showed no histopathological alteration. The typical histological structure of the glomeruli and tubules at the cortex was recorded in (**A**). Photomicrographs (**B**–**E**): Transverse renal tissue section from AlCl_3_-treated animals showing focal inflammatory cell infiltration between the basophilic dysplastic renal tubules (**B**–**D**). Eosinophilic cast formation was detected in the lumen of some flattened lining epithelium tubules (**E**) (arrows). Photomicrographs (**F**,**G**): Transverse renal tissue section from the fluoxetine-treated animals showing inflammatory cell infiltration, and fibrosis with edema was observed in the capsule (**F**). There were focal hemorrhages between the tubules associated with congestion in the blood vessels at the cortex (**G**) (arrows). Photomicrograph (**H**): Transverse renal section from the wheatgrass group showing congestion in the cortical blood vessels (**H**) (arrows). Photomicrograph (**I**): Transverse renal tissue section from the combination group showing no histopathological alteration as recorded in (**I**).

**Figure 4:**
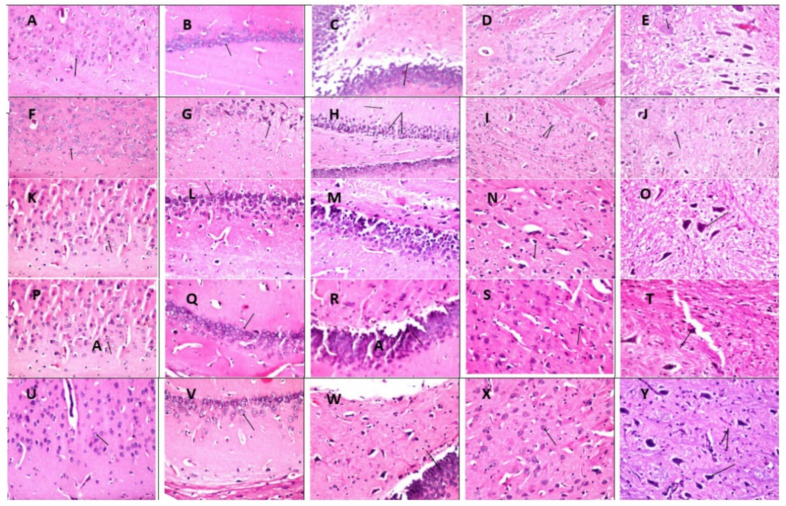
Photomicrographs of brain tissue specimens stained by H & E (×40). Photomicrographs (**A**–**E**): Transverse brain tissue sections from the control group showing no histopathological alteration in the cerebral cortex, hippocampus, striatum, or substantia nigra. Photomicrographs (**F**–**J**): Transverse brain tissue sections from AlCl_3_-treated animals showing no histopathological alteration in the cerebral cortex (**F**). The pyramidal cells in the hippocampus showed nuclear pyknosis and degeneration as well as in the fascia dentate, while the neurons in the subiculum were intact (**G**,**H**). There was congestion in the blood vessels of the striatum (**I**). Atrophy was detected in some of the neurons in substantia nigra (**J**) (arrows). Photomicrographs (**K**–**O**): The transverse brain tissue section from the fluoxetine-treated animals shows nuclear pyknosis and degeneration in most cerebral cortex neurons (**K**). The subiculum in the hippocampus was intact (**L**), while the fascia dentate showed nuclear pyknosis in a few neuronal cells (**M**) and gliosis in the striatum (**N**). There was no histopathological alteration in the substantia nigra (**O**) (arrows). Photomicrographs (**P**–**T**): Transverse brain tissue section from the wheatgrass group showing nuclear pyknosis in some few neurons at the cerebral cortex (**P**). The subiculum in the hippocampus showed typical histological structure (**Q**), while the fascia dentate had nuclear pyknosis in a few neurons (**R**). Diffuse gliosis was detected in the striatum (**S**), while the substantia nigra was intact (**T**) (arrows). Photomicrographs (**U**–**Y**): Transverse brain tissue section from the combination group showing cerebral cortex and subiculum in the hippocampus with typical histological structure, while the fascia dentata showed nuclear pyknosis in few neurons (**U**–**W**). Diffuse gliosis was detected in the striatum (**X**), as well as substantia nigra (**Y**) (arrows).

The findings were shown in Figure 2, Figure 3 and Figure 4, which illustrated the magnitude of histopathological alterations in tissue specimens from various experimental groups as illustrated in each figure legend.

## 3. Discussion

Due to its low price, aluminum cookware is still used in many Egyptian kitchens, especially in rural areas, as well as in many other developed countries. Our study focused on the effects of aluminum on mental health and its consequences on the liver and the kidneys and the possible role of wheatgrass juice alone or in combination with fluoxetine in attenuating these effects.

In our experiments, the AlCl_3_ group was injected daily with AlCl_3_ (70 mg/kg i.p) for five successive weeks; the results of these groups revealed induction of AD, which was proved by behavioral testing (MWT).

Regarding histological evaluation, exposure to AlCl_3_ caused severe and noticeable histopathological alterations in the hippocampus, including neuronal degeneration in various regions of the hippocampus and cytoplasmic vacuolization, hemorrhage, and gliosis was identical to a previous study [30]. As it contains glutamatergic, monoaminergic, and cholinergic axon terminals, the hippocampus is the earliest and most afflicted area in the brain in AD [31].

AlCl_3_ is implicated in metabolic and redox processes in the central nervous system, and its presence in the blood is linked to an increased risk of AD [32]. The results of this work showed a significant alteration in various brain biochemical parameters in the AlCl_3_ group when compared to the control group. There is a significant elevation in the levels GSK-3β, TAU, Aβ, and ACHE and a significant decline in β-catenin, BDNF, and neurotransmitter (DA, 5HT, and NE). Oxidative stress status and inflammation were noticed, characterized by a significant decrease in SOD and TAC with a significant increase in MDA, IL-1β, and TNF-α. These findings are inconsistent with previous studies [33,34,35,36].

GSK-3β and β-catenin are markers of the Wnt/β-catenin signaling pathway. Wnt/-catenin signaling is downregulated in the aging brain, leading to diminished neurogenesis and cognitive decline [37]. Wnt/-catenin signaling is an essential signaling system that controls a variety of biological activities, including cell survival. GSK-3β is thought to represent a crucial molecular link between senile plaques and neurofibrillary tangles, two histological hallmarks of AD. Two effects follow the elevation in GSK-3β, the first of them is TAU hyperphosphorylation which leads to neurofibrillary tangles and neuronal death; the second effect is β-catenin degradation which in turn induces Aβ formation [38,39].

Furthermore, GSK-3β is thought to play a function in choline metabolism, including the control of ACHE [40]. ACHE activity in the brain plays a crucial role in maintaining normal brain function. Chronic exposure to AlCl_3_ could elevate lipid peroxidation caused by free radical and ROS formation, leading to ACHE activity and decreased acetylcholine, affecting cognition and memory [41].

It is established that proinflammatory cytokines, particularly IL-1β, can downregulate BDNF expression in cognition-related brain regions, including the hippocampus [42]. BDNF has essential physiological activities in both the peripheral and central nervous systems [43]. BDNF can promote the survival, growth, differentiation, and development of neurons and plays a crucial role in the neural structure and functional plasticity. There is mounting evidence that alterations in cerebral BDNF levels and the BDNF-TrkB signaling pathway may play a role in the etiology of AD [44].

As neuroinflammation is known to influence multiple BDNF-related signaling pathways, a current theory posits that these low BDNF levels may be attributable to the chronic inflammatory state of the brain in AD. Glia cell activation can increase pro-and anti-inflammatory cytokines and reactive oxygen species, contributing to neuronal function modification and neurotoxicity, as seen in various brain diseases [45].

TNF also has damaging consequences on synaptic transmission and plasticity, such as long-term activation and synaptic scaling, essential in learning and memory [46]. During old age, injury, and various disease states, TNF can become damaging and even poisonous. TNF, like most cytokines, is relatively low in a healthy adult brain but quite high in neurodegenerative brains, and neuroinflammation can be diagnosed years before neuronal cell death [47].

Furthermore, aluminum neurotoxicity disturbs cerebrospinal fluid tetrahydrobiopterin levels, which cause a drop in brain neurotransmitters levels [36]. This drop is linked to decreased BDNF levels, which have trophic effects on serotonergic and catecholaminergic neurons [48,49,50].

On the other hand, the consequences of AlCl_3_ intake in the experimental group revealed a marked deterioration and toxicity in hepatic and renal tissues. A histological examination revealed several detrimental morphological abnormalities in the renal and hepatic tissues of the AlCl_3_ group in the current investigation.

Our results showed a significant increase in serum ALT, AST, ALP, creatinine, and urea levels as well as oxidative stress, inflammatory status, and apoptosis in both hepatic and renal tissue marked by a significant increase in the levels of IL-6, TNF-α, NF-κB, NO, MDA, and caspase 3 activity with a significant decrease in the levels of SOD, and TAC. Furthermore, we found a significant increase in serum TC and TAG levels and a significant decrease in HDL levels. These findings are in agreement with previous studies [51,52,53,54,55].

Hepatotoxicity and nephrotoxicity may be caused by AlCl_3_ buildup in tissues. Higher liver and kidney function indicators are the predominant signs of AlCl_3_ hepatotoxicity and nephrotoxicity [54,56]. Exposure to AlCl_3_ produces liver necrosis, significant cellular membrane damage, and subsequent release of intracellular enzymes and bilirubin into the bloodstream [51]. The possible mechanisms of kidney–brain crosstalk in terms of inflammatory molecules are premised on the reality that cytokines, including IL-1β, IL-6, TNF-α, which are frequently implicated in the formation of CKD, can influence distant organs like the brain [55].

Another critical aspect of the inflammatory hypothesis is oxidative stress and its damaging and pathogenic potential through the distortion of proteins, lipids, nucleic acids, organ dysfunction, and apoptosis [57]. Aberrant lipid metabolism has been associated with an increased risk of developing AD, as the liver is the principal peripheral organ responsible for lipid metabolism [58].

The hepatotoxicity and nephrotoxicity may worsen AD’s status through the inability to maintain Aβ homeostasis at the periphery, as they play an essential role in the elimination of Aβ from the circulation [15,17].

Our study used fluoxetine, wheatgrass, and their combination in the treatment groups to explore their possible roles in alleviating AD symptoms and attenuating its consequences on liver and kidney tissues.

The results of our work showed a significant alleviation in all measured brain biomarkers revealed by an improvement in oxidative stress and inflammatory status, behavioral test, histopathological examination, and brain Wnt/-catenin pathway markers. The best improvement was noticed in the combination group, followed by the fluoxetine group, and then the wheatgrass group. The improvement in cognition and memory is accompanied by improved liver and kidney function and their oxidative stress and inflammatory status.

Fluoxetine is a selective serotonin reuptake inhibitor antidepressant that could improve memory and cognitive function and relieve depression and anxiety among AD patients. Fluoxetine has been shown to be able to inhibit β-amyloid production and prevent neuronal degeneration as it could restore Wnt/β-catenin signaling by enhancing GSK-3β phosphorylation and increasing the β-catenin level [20,21,22].

Wheatgrass can restore antioxidant levels such as SOD, catalase, and reduced glutathione [59]. Wheatgrass can prevent and fix free radical damage to liver cells. Chlorophyll seems to be the most crucial component in the process. Chlorophyll is swiftly absorbed into the bloodstream and aids in liver cleansing, blood sugar and blood flow improvement, immune system building, and detoxification [60]. Wheatgrass juice enhanced kidney function markers (urea, creatinine, and uric acid). It contains vitamin C and phenolic compounds, which induced liver and kidney organ detoxification and blood purification of pollutants [61].

TNF-α, IL-1β, and IL-6 are proinflammatory cytokines that cause liver damage via modulating the oxidative state and ultimately organ destruction [62]. Endotoxin shock is mediated by TNF-α, which also promotes caspase-dependent apoptotic signaling [63]. Excessive formation of ROS initiates hazardous oxidative events, and NF-κB activates various genes associated with an oxidative state, such as glutathione peroxidase and SOD [64]. Wheatgrass components have been demonstrated to have antioxidant and anti-inflammatory effects via inhibiting NF-κB activation in previous research [65]. Wheatgrass dramatically reduced the generation and release of these inflammatory mediators, according to our findings.

Our finding revealed that wheatgrass was able to restore Wnt/-catenin signaling, inhibit GSK3β, which in turn increased the level of β-catenin. The result of this pathway is inhibiting the formation of Aβ plaque and the protection from NFTs.

## 4. Materials and Methods

### 4.1. Animals

Fifty adult male Wistar rats weighing approximately 280–320 g were used in the current study. Rats were obtained from the Nile Co. for Pharmaceuticals and Chemical Industries, Cairo, Egypt. They were housed in stainless steel cages, three to four per cage, at a temperature of 25 ± 1 °C with alternate 12 h light and dark cycles. Rats were kept under the same suitable conditions and provided with their daily dietary requirements of standard diet pellets (El-Nasr, Abu Zaabal, Cairo, Egypt) containing not less than 20% protein, 5% fiber, 3.5% fat, 6.5% ash, and a vitamin mixture; water was given ad-libitum.

### 4.2. Ethical Statement

The protocol of this study was approved by the “Al-Azhar University—Institutional Animal Care and Use Committee” (297/2020). All efforts were made to diminish the distress of rats during the entire experimental period.

### 4.3. Drugs and Chemicals

Wheatgrass powder was purchased from Bioglan Superfoods (Surrey, England, UK), fluoxetine HCl (CAS#:56296-78-7), and aluminum chloride hydrated (AlCl_3_. 6H_2_O, CAS#:7784-13-6) were purchased from the Sigma Chemical Co. (St. Louis, MO, USA). All other chemicals and materials were commercially available and of high quality.

The wheatgrass solution was prepared by dissolving 1 gm of wheatgrass powder in 10 mL distilled water, kept for 6 h, and shaken well before oral administration [66]. AlCl_3_ solution was prepared by dissolving 20 mg AlCl_3_ in 1 mL distilled water and was adjusted to pH 7.4 with 0.1 M phosphate buffer saline [67]. Fluoxetine HCl was dissolved in a distilled equivalent to 10 mg/mL [68].

### 4.4. Experimental Design

The rats were divided into five groups (*n* = 10) and assigned to different treatments for five weeks. Group 1 served as a control and was given distilled water daily. Group 2 was injected daily with AlCl_3_ (70 mg/kg i.p) [69]. AD rats in Group 3 were received fluoxetine (10 mg/kg, p.o) [70]. AD rats in Group 4 were treated with Wheatgrass (100 mg/kg, p.o) [71]. AD rats in Group 5 were injected with a combination of fluoxetine (10 mg/kg, p.o) and wheatgrass (100 mg/kg, p.o).

### 4.5. Behavioral Study (Morris Water Maze (MWM) Test)

A behavioral study was conducted between 8:00 AM and 4:00 PM at standard laboratory conditions. The MWM test was carried out to examine memory and spatial learning. The MWM included a circular pool (Zhenghua Bio Instruments Ltd., Huaibei, China), an analysis system, and an automatic camera [72]. The pool (1.2 m in diameter) was full of non-toxic opaque water to a deepness of 50 cm. The water temperature was adjusted to 23 ± 2 °C. The MWM was divided into four equal quadrants, and four different equidistant visual cues were placed on the inner wall of the pool for mouse positioning. The cylindrical escape platform (12 cm in diameter) was placed in the center of a designated quadrant with its top 1 cm below the water surface.

After four days of environmental adaptation, the rats were first trained for five consecutive days on spatial learning. In the hidden-platform test, each rat received four trials per day to find the submerged platform at a fixed quadrant center, and escape latencies were recorded as the arithmetic means of the four trials. In each training unit, the rat was placed into the water facing the pool wall and allowed to swim freely to the escape platform. After reaching the platform, the rat was allowed to stay there for 5 s. If it failed to find the platform within 60 s, the rat was manually guided and allowed to remain on it for 30 s. The rat was subsequently returned to the home cage for 60 s before the subsequent trial. A probe test for spatial memory was conducted on day four. The platform was removed, and the swimming time was limited to 60 s. The escape latency (s) and the time spent in the target quadrant were recorded and analyzed [73].

### 4.6. Sample Preparation & Measurments

At the end of the 5th week, 24 h. after the behavior test, fasted rats were anesthetized. Blood samples were collected via eye puncture from each rat before scarification into serum separator tubes, allowed to stand (30 min), centrifuged (3000 rpm for 15 min), serum collected and stored at −80 °C until the assay of the studied biochemical parameters.

Rats were sacrificed, and the brains, livers, and kidneys were dissected and washed with ice-cold saline. The whole-brain tissues were divided into two parts, one for histopathological examination, and the other part was immediately homogenized to give 10% (*w*/*v*) homogenate in an ice-cold medium containing 50 mM Tris-HCl (pH 7.4) and 300 mM sucrose [74]. The liver and kidneys were immediately rinsed with ice-cold saline and dried; tissues were homogenized. The homogenate was centrifuged at 4000 rpm for 10 min at 4 °C [51].

The sera were used for the determination of liver functions (alanine transaminase [ALT], aspartate transaminase [AST], and alkaline phosphatase [ALP]), kidney functions (urea and creatinine), and lipid profile (total cholesterol [TC], high-density lipoprotein [HDL] and triacylglycerol [TG]).

The brain, liver, and kidney homogenates were used for the determination of oxidative stress markers (total antioxidant capacity [TAC], nitric oxide [NO], superoxide dismutase [SOD] and malondialdehyde [MDA]), and tumor necrosis factor-α (TNF-α). Furthermore, the kidney and liver homogenate were used for the determination of interleukin-6 (IL-6), nuclear factor kappa B (NF-κB), as well as Caspase-3 activity.

The brain homogenate was used for the assessment of β-Catenin and Glycogen synthase kinase-3 beta (GSK-3β) activity, Brain monoamines neurotransmitters [dopamine (DA), serotonin (5-HT) and norepinephrine (NE)], proinflammatory brain interleukin-1β (IL-1β), Aβ, tau protein (TAU), acetylcholine esterase (ACHE), and BDNF.

#### 4.6.1. Estimation of Hepatic and Renal Functions

Assessments of serum levels of AST, ALT, ALP, urea, and creatinine were carried out using a commercial kit supplied by Spinreact (Sant Colom, Spain) ref No. 41270, 41280, 1001130, 1001329, and 1001110, respectively.

#### 4.6.2. Estimation of Lipid Profile

Colorimetric assay kits supplied by Spinreact (Sant Colom, Spain) were used for the measurement of serum TC, HDL, and TG ref No. 1001090, 1001095, and 1001310, respectively.

#### 4.6.3. Assessment of Apoptosis and Inflammatory Mediators

TNF-α in the brain, liver and kidney tissues’ homogenate was assayed using the commercially available Quantikine^®^ Rat TNF-α ELISA Kit (catalog No. RTA00, R&D Systems, Minneapolis, MN, USA). NFκ-B and IL-6 were assayed in the renal and hepatic homogenate using NFκ-B ELISA kit Cusabio Biotech (code: CSB-E13148r, Cusabio Life Science, Inc., Wuhan, Hubei Province, China) and RayBio^®^ Rat IL-6 (code: ELR-IL6-1, RayBiotech, Inc., Peachtree Corners, GA, USA), respectively.

Caspase-3 activity was detected in the renal and hepatic homogenates using Caspase 3 Assay Colorimetric Kit Catalog No. CASP3C, supplied by the Sigma-Aldrich Co. (St. Louis, MO, USA).

IL-1β was measured in brain tissue homogenate using an ELISA kit supplied by Cusabio Biotech (code: CSB-E08055r, Cusabio Life Science, Inc., Wuhan, Hubei Province, China).

#### 4.6.4. Brain, Hepatic, and Renal Oxidative Stress Markers

Thiobarbituric acid reactive substances measured as MDA, NO estimation, SOD activity, and TAC level were measured using colorimetric assay kits supplied by (Biodiagnostic, Cairo, Egypt).

#### 4.6.5. Determination of β-Catenin, GSK-3β in Brain Tissue

GSK-3β content was detected in brain tissue homogenate using ELISA kit (catalog No. CBEL-GSK3b-1) supplied by RayBiotech, Peachtree Corners, GA, USA. Besides, β-Catenin content was detected in brain tissue homogenate using ELISA kit (catalog No. K3383-100) provided by BioVision, Milpitas, CA, USA.

#### 4.6.6. Determination of Brain Monoamines

Immediately after sacrificing the rats, concentrations of brain monoamines were detected because changes in the level of brain monoamines may occur within a few minutes [75]. DA, NE, and 5-HT were assayed using the ELISA method using an assay kit purchased from MyBioSource, Inc., San Diego, CA, USA. Product Number MBS7214676, MBS269993, and MBS2611553, respectively.

#### 4.6.7. Determination of Beta-Amyloid (Aβ) Content in Brain Tissue

Aβ content was measured in brain tissue homogenate using ELISA kit number (MBS702915) supplied by My Bio Source, Inc., San Diego, CA, USA.

#### 4.6.8. Determination of Tau Protein (TAU) in Brain Tissue

Tau protein content was measured in brain tissue homogenate using ELISA kit number (MBS725098) was used for this detection and supplied by MyBioSource, Inc., San Diego, CA, USA.

#### 4.6.9. Determination of ACHE in Brain Tissue

ACHE content was detected in brain tissue homogenate using a colorimetric assay kit (catalog no. MAK119) supplied by Sigma-Aldrich Co. (St. Louis, MO, USA).

#### 4.6.10. Determination of Brain-Derived Neurotrophic Factor (BDNF) in Brain Tissue

Content of BDNF in brain tissue homogenate was assayed by ELISA method using assay kit purchased from MyBioSource, Inc., San Diego, CA, USA. Product Number MBS494147.

#### 4.6.11. Histopathological Examination of Brain Tissue and Liver and Kidney

Brain, liver, and kidney tissue samples were fixed in 10% formalin for 24 h and subsequently washed with water and a serial dilution of alcohol for dehydration. Specimens were embedded in paraffin and sectioned by microtome to 4 microns thickness. Afterward, the obtained tissue sections were collected on glass slides for deparaffinization, stained with hematoxylin and eosin stain for the routine histological examination using light microscopy [76].

### 4.7. Statistical Analysis

Data are presented as mean ± SD. Multiple comparisons were performed using one-way ANOVA followed by Tukey Kramer as a post hoc test. Statistical analysis and graphs were performed using Graph Pad Prism (ISI^®^, San Diego, CA, USA) software (version 5).

## 5. Conclusions

Fluoxetine and *Triticum aestivum* have an ameliorative effect on aluminum-induced AD in rats. They have a neuroprotective impact as they can restore cognitive deficits, increase acetylcholinesterase enzyme activity and monoamine levels, prevent neuronal degeneration, and reduce oxidative stress and inflammation. In addition, they alleviate anomalies that arise in the liver or kidneys at this time, which may increase their vulnerability to AD. Furthermore, the combination of fluoxetine and *Triticum aestivum* demonstrated more significant effects in treating AD than fluoxetine alone. To confirm these beneficial results, further clinical studies in aged people are required to determine the exact dose of fluoxetine and wheatgrass.

## Figures and Tables

**Table 1:** Effect of fluoxetine, wheatgrass, or their combination on learning and memory performance in rats.

MWM	“Time Spent (sec) in Target Quadrant”	“Escape Latency (sec) for Total Four Days”
Control	50.80 ± 1.43	64.93 ± 1.06
AD	17.00 ^a^ ± 1.14	147.7 ^a^ ± 2.96
AD/Fluoxetine	34.80 ^abcd^ ± 1.74	79.02 ^abc^ ± 0.37
AD/Wheatgrass	39.20 ^abd^ ± 0.86	97.03 ^abd^ ± 0.48
AD/Combination	46.20 ^bc^ ± 0.86	78.08 ^bc^ ± 0.60

Number of animals in each group (*n* = 10). Data were expressed as means ± SD. ^a, b, c,^ or ^d^; Significantly different from the control, AD, wheatgrass/AD, or combination/AD group, respectively, *p* < 0.05 using ANOVA followed by Tukey–Kramer as post hoc test.

**Table 2:** Effect of fluoxetine, wheatgrass, or their combination on serum alanine aminotransferase (ALT), aspartate aminotransferase (AST), alkaline phosphatase (ALP), creatinine, urea, total cholesterol (TC), triglycerides (TG), and high-density lipoprotein (HDL).

Serum	Control	AD	AD/Fluoxetine	AD/Wheatgrass	AD/Combination
ALT (U/L)	14.13 ± 0.56	97.83 ^a^ ± 0.78	70.63 ^abcd^ ± 1.57	39.63 ^abd^ ± 0.59	30.63 ^abc^ ± 0.59
AST (U/L)	21.10 ± 1.04	92.03 ^a^ ± 0.73	53.38 ^abcd^ ± 1.45	42.83 ^abd^ ± 1.83	31.78 ^abc^ ± 0.59
ALP (U/L)	114.61 ± 0.39	372.80 ^a^ ± 3.05	198.72 ^abcd^ ± 0.97	186.04 ^abd^ ± 1.47	166.03 ^abc^ ± 7.91
Creatinine (mg/dL)	0.29 ± 0.01	4.03 ^a^ ± 0.21	2.85 ^abcd^ ± 0.03	1.80 ^abd^ ± 0.15	0.76 ^bc^ ± 0.01
Urea (mg/dL)	45.13 ± 1.89	90.20 ^a^ ± 0.25	63.56 ^abcd^ ± 0.84	49.36 ^b^ ± 1.81	45.38 ^b^ ± 1.48
TC (mg/mL)	132.72 ± 1.85	301.90 ^a^ ± 2.51	206.04 ^abcd^ ± 1.47	175.63 ^ab^ ± 1.57	171.90 ^ab^ ± 2.95
TG (mg/mL)	72.05 ± 1.42	135.21 ^a^ ± 1.56	85.06 ^abcd^ ± 1.16	79.40 ^ab^ ± 0.32	76.42 ^b^ ± 0.65
HDL (mg/mL)	63.94 ± 0.85	18.93 ^a^ ± 0.66	40.48 ^abd^ ± 0.70	41.81 ^abd^ ± 0.27	48.85 ^abc^ ± 0.67

Number of animals in each group (*n* = 10). Data were expressed as means ± SD. ^a b, c,^ or ^d^: Significantly different from the control, AD, wheatgrass/AD, or combination/AD group, respectively, *p* < 0.05 using ANOVA followed by Tukey–Kramer as a post hoc test.

**Table 3:** Effect of fluoxetine, wheatgrass, or their combination on hepatic interleukin-6 (IL-6), tumor necrosis factor-α (TNF-α), nuclear factor kappa B (NF-κB), caspase-3 activity, malondialdehyde (MDA), nitric oxide (NO), superoxide dismutase (SOD), and total antioxidant capacity (TAC).

Hepatic	Control	AD	AD/Fluoxetine	AD/Wheatgrass	AD/Combination
IL-6 (pg/mg)	31.45 ± 0.37	131.92 ^a^ ± 0.64	90.22 ^abcd^ ± 1.59	77.93 ^abd^ ± 0.72	61.62 ^abc^ ± 0.36
TNF-α (pg/mg)	32.67 ± 1.27	120.93 ^a^ ± 0.41	98.34 ^abcd^ ± 0.67	83.56 ^abd^ ± 0.84	63.03 ^abc^ ± 2.43
NF-κB (pg/mg)	1.00 ± 0.01	9.98 ^a^ ± 0.06	6.19 ^abcd^ ± 0.14	4.695 ^ab^ ± 0.11	2.38 ^abc^ ± 0.07
Caspase-3 Activity (µM pNA/min/mL)	1.98 ± 0.03	5.06 ^a^ ± 0.02	3.72 ^abcd^ ± 0.06	2.78 ^ab^ ± 0.03	2.72 ^ab^ ± 0.06
SOD (U/mg)	2.96 ± 0.04	0.39 ^a^ ± 0.02	1.01 ^abcd^ ± 0.02	1.65 ^abd^ ± 0.12	2.22 ^abc^ ± 0.08
MDA (mmol/g)	11.25 ± 0.41	104.91 ^a^ ± 2.70	88.58 ^abcd^ ± 0.76	52.48 ^abd^ ± 0.81	28.94 ^abc^ ± 1.33
TAC (nmol/mg)	27.98 ± 0.44	9.32 ^a^ ± 0.34	14.96 ^abcd^ ± 0.10	11.88 ^abd^ ± 0.17	21.28 ^abc^ ± 0.44
NO (nmol/mg)	1.63 ± 0.04	20.30 ^a^ ± 0.58	12.43 ^abcd^ ± 0.53	7.94 ^abd^ ± 0.16	6.10 ^abc^ ± 0.05

Number of animals in each group (*n* = 10). Data were expressed as means ± SD. ^a b, c,^ or ^d^: Significantly different from the control, AD, wheatgrass/AD, or combination/AD group, respectively, *p* < 0.05 using ANOVA followed by Tukey–Kramer as post hoc test.

**Table 4:** Effects of fluoxetine, wheatgrass, or their combination on renal interleukin-6 (IL-6), tumor necrosis factor-α (TNF-α), nuclear factor kappa B (NF-κB), caspase-3, malondialdehyde (MDA), nitric oxide (NO), superoxide dismutase (SOD), and total antioxidant capacity (TAC).

Renal	Control	AD	AD/Fluoxetine	AD/Wheatgrass	AD/Combination
IL-6 (pg/mg)	33.50 ± 2.15	99.06 ^a^ ± 1.23	66.49 ^abcd^ ± 1.55	56.36 ^ab^ ± 1.27	51.58 ^ab^ ± 0.66
TNF-α (pg/mg)	36.43 ± 0.22	135.6 ^a^ ± 1.41	84.79 ^abc^ ± 1.68	85.13 ^abd^ ± 1.29	71.91 ^abc^ ± 0.33
NF-κB (pg/mg)	1.03 ± 0.04	4.69 ^a^ ± 0.05	3.17 ^abc^ ± 0.09	2.99 ^abd^ ± 0.02	2.69 ^abc^ ± 0.05
Caspase-3 Activity (µM pNA/min/mL)	2.87 ± 0.06	19.36 ^a^ ± 0.27	7.98 ^abc^ ± 0.14	8.12 ^abd^ ± 0.17	7.09 ^abc^ ± 0.24
SOD (U/mg)	2.36 ± 0.09	0.28 ^a^ ± 0.01	0.78 ^abc^ ± 0.03	0.92 ^abd^ ± 0.01	1.35 ^abc^ ± 0.11
MDA (mmol/g)	6.68 ± 0.04	44.66 ^a^ ± 1.23	27.23 ^abcd^ ± 1.32	16.71 ^abd^ ± 0.78	11.08 ^abc^ ± 0.56
TAC (nmol/mg)	29.60 ± 0.50	12.3 ^a^ ± 0.21	16.70 ^abcd^ ± 0.32	18.72 ^abd^ ± 0.14	21.83 ^abc^ ± 0.56
NO (nmol/mg)	0.94 ± 0.03	11.65 ^a^ ± 0.37	8.17 ^abcd^ ± 0.09	5.12 ^abd^ ± 0.17	2.99 ^abc^ ± 0.03

Number of animals in each group (*n* = 10). Data were expressed as means ± SD. ^a b, c,^ or ^d^. Significantly different from the control, AD, wheatgrass/AD, or combination/AD group, respectively, *p* < 0.05 using ANOVA followed by Tukey–Kramer as post hoc test.

**Table 5:** Effect of fluoxetine, wheatgrass, or their combination on cerebral β-catenin, glycogen synthase kinase-3 (GSK-3β), dopamine (DA), norepinephrine (NE), serotonin (5-HT), interleukin 1β (IL-1β), and tumor necrosis factor-α (TNF-α), total antioxidant capacity (TAC), superoxide dismutase (SOD), and malondialdehyde (MDA).

Cerebral	Control	AD	AD/Fluoxetine	AD/Wheatgrass	AD/Combination
β –Catenine (nmol/mg)	3.19 ± 0.07	0.61 ^a^ ± 0.07	1.78 ^abcd^ ± 0.06	2.50 ^ab^ ± 0.03	3.13 ^b^ ± 0.09
GSK-3β (nmol/mg)	1.01 ± 0.01	10.12 ^a^ ± 0.10	5.78 ^abc^ ± 0.29	5.65 ^abd^ ± 0.21	3.75 ^abc^ ± 0.14
DA (nmol/mg)	68.18 ± 0.91	16.24 ^a^ ± 0.51	38.76 ^abcd^ ± 0.82	28.28 ^abd^ ± 0.54	45.14 ^abc^ ± 0.01
NE (nmol/mg)	721.1 ± 2.66	236.1 ^a^ ± 0.47	584.1 ^abcd^ ± 3.85	451.1 ^abd^ ± 5.05	591.2 ^abc^ ± 2.91
5-HT (nmol/mg)	11.56 ± 0.08	4.06 ^a^ ± 0.05	9.03 ^abcd^ ± 0.51	6.85 ^abd^ ± 0.08	10.52 ^bc^ ± 0.31
IL-1β (pg/mg)	28.20 ± 0.43	117.94 ^a^ ± 1.01	57.57 ^abcd^ ± 2.96	83.33 ^abd^ ± 3.04	54.03 ^abc^ ± 0.86
TNF-α (pg/mg)	27.02 ± 0.04	212.13 ^a^ ± 4.36	63.18 ^abcd^ ± 0.45	87.53 ^abd^ ± 0.56	59.43 ^abc^ ± 2.03
SOD (U/mg)	3.62 ± 0.04	0.32 ^a^ ± 0.02	1.78 ^abcd^ ± 0.06	2.31 ^abd^ ± 0.06	2.78 ^abc^ ± 0.04
MDA (mmol/g)	6.46 ± 0.19	99.10 ^a^ ± 3.56	36.07 ^abcd^ ± 1.87	45.38 ^abd^ ± 2.02	31.73 ^abc^ ± 1.49
TAC (nmol/mg)	32.78 ± 0.76	9.15 ^a^ ± 0.45	18.55 ^abc^ ± 0.15	17.73 ^abd^ ± 1.01	22.32 ^abc^ ± 0.69

Number of animals in each group (*n* = 10). Data were expressed as means ± SD. ^a b, c,^ or ^d^; Significantly different from the control, AD, wheatgrass/AD, or combination/AD group, respectively, *p* < 0.05 using ANOVA followed by Tukey–Kramer as post hoc test.

## Data Availability

The data presented in this study are available upon request from the corresponding author.

## Sample Availability

Samples of the compounds are available from the authors.
